# Supplementary material for: A new view on the morphology and phylogeny of eugregarines suggested by the evidence from the gregarine Ancora sagittata (Leuckart, 1860) Labbé, 1899 (Apicomplexa: Eugregarinida)
Source: PeerJ. 2017 May 30;5:e3354. doi: 10.7717/peerj.3354 (PMC5452951; doi:10.7717/peerj.3354)
Supplement: Supplemental Information 11 — Direct pairwise comparison of Ancora sagittata sequences with each other (separately, SSU rDNA alone and concatenated SSU, 5.8S, and LSU rDNAs) and of A. sagittata and Polyplicarium spp. SSU rDNA sequences. The lengths of the overlaps in the alignment, mismatches (substitutions and indels), and percentage of identity of overlapping regions are shown (1st, 2nd, and 3rd lines of the cells, respectively). The diagonal of the table indicates the sequence total lengths. For A. sagittata sequences, two values are given: for SSU rDNA alone and for complete contigs without external spacers (ETS). [file peerj-05-3354-s011.doc]

Supplementary Table 1. Direct pairwise comparison of *Ancora sagittata* sequences with each other (separately, SSU rDNA alone and concatenated SSU, 5.8S, and LSU rDNAs) and of *A. sagittata* and *Polyplicarium* spp. SSU rDNA sequences. The lengths of the overlaps in the alignment, mismatches (substitutions and indels), and percentage of identity of overlapping regions are shown (1^st^, 2^nd^, and 3^rd^ lines of the cells, respectively). The diagonal of the table indicates the sequence total lengths. For *A. sagittata* sequences, two values are given: for SSU rDNA alone and for complete contigs without external spacers (ETS).

|  | *A. sagittata* Roscoff | *A. sagittata* WSBS 2006 | *A. sagittata* WSBS 2010 | *A. sagittata* WSBS 2011 | *Polyplicarium*  *citrusae* | *Polyplicarium*  *lacrimae* | *Polyplicarium*  *curvarae* | *Polyplicarium*  *translucidae* |
| --- | --- | --- | --- | --- | --- | --- | --- | --- |
| *A. sagittata* Roscoff 2009  contig (ribosomal operon)  KX 982501 | 4847 |  |  |  |  |  |  |  |
| *A. sagittata* WSBS 2006  contig (ribosomal operon)  KX982502 | 1688/2628  1/18  99.9/99.3% | 2633 |  |  |  |  |  |  |
| *A. sagittata* WSBS 2010  contig (ribosomal operon)  KX982504 | 1709/4849  21/185  98.8/96.2% | 1689/2633  22/151  98.7/94.3% | 5973 |  |  |  |  |  |
| *A. sagittata* WSBS 2011  contig (ribosomal operon)  KX982503 | 1709/4850  0/31  100/99.4% | 1689/2633  1/8  99.9/99.7% | 1736/5281  25/202  98.6/96.2% | 6961 |  |  |  |  |
| *Polyplicarium citrusae*  (SSU rDNA alone))  JX535336 | 1732  282  83.7% | 1712  284  83.4% | 1737  282  83.8% | 1737  283  83.7% | 1725 |  |  |  |
| *Polyplicarium lacrimae*  (SSU rDNA alone)  JX535336 | 1629  238  85.4% | 1610  240  85.1% | 1639  234  85.7% | 1639  239  85.4% | 1637  242  85.2% | 1629 |  |  |
| *Polyplicarium curvara*  (SSU rDNA alone)  JX535340 | 1636  213  87% | 1637  215  86.9% | 1637  219  86.6% | 1637  214  86.9% | 1641  194  88.2% | 1596  179  88.8% | 1629 |  |
| *Polyplicarium translucidae*  (SSU rDNA alone)  JX535348 | 1561  200  87.2% | 1562  202  87.1% | 1562  201  87.1% | 1562  201  87.1% | 1568  215  86.3% | 1518  151  90.1% | 1562  140  91% | 1553 |
